# Epilepsy

**Published:** 1893-07

**Authors:** Joseph Heinrich


### EPILEPSY.

Epilepsy, or "falling sickness," is the sudden loss of all consciousness with convulsions, foaming at the mouth, or livid face, with utter prostration of power and sense; in a few minutes the patient recovers, but without the slightest recollection of what has taken place. These attacks come on, apparently, as sudden and as unanticipated as a stroke of lightning in a clear sky. The original word means "to seize upon," as at any time, in conversing with a friend, or seated at the table, or riding in a carriage, or sitting by the fire, and with every external appearance of perfect health, these fits come on, with fearful contortions, with grinding of teeth and uncontrollable action of every limb and muscle of the body. It is most generally an incurable disease of the brain as a result of a scrofulous constitution. This epileptic condition, or susceptibility, may be in a person, but may never be brought out, never developed because an exciting cause may never be applied, just as powder will never explode unless a spark is applied. The object of this article is mainly to state some of the exciting causes of epilepsy, and thus prevent the development of so unfortunate a habit of body; for its nature is such, that if it occurs but a few times, the habit is formed for a lifetime, or an exemption is purchased only at the price of an eternal and painful vigilance.

The epileptic habit is nearly always set up in early childhood, the most common causes being terror or sudden fright, such as may be occasioned by some sudden noise or the presentation of some terrifying object. It is not always that the child survives the first fit, and pity is it that it ever should, for it is nothing short of a living crucifixion to a parent's heart to witness the terrible contortions which *seem* to rack, with unendurable agony, every fibre of the innocent and uncomplaining sufferer; we say *seem* with an emphasis, for every circumstance connected with an epileptic attack indicates, most unmistakably, an utter unconsciousness of any bodily suffering. A child under three years of age was left in charge of a nurse, while the mother attended an evening party. On repairing to its little crib, on her return, to see that all was well after the assurance of the maid that it had been sleeping soundly, not having made the "slightest bit of a noise," the eyes were glaring widely open, the whole features were stamped with an expression of vague and indescribable horror, and life was extinct. At the feet of the child had been placed a human skull taken from a doctor's office table.

Parents sometimes frighten their children for the amusement of witnessing their gestures and exclamations; as to its reprehensibility, we need make no remarks.

When an epileptic attack is repeated two, three, or four times, there is seldom any refuge short of the grave, the end being fatality or sudden death. Our greatest anxiety in this article is to attract parental attention to the *first attack*, so that, by exercising a most untiring vigilance against the causes which may repeat it, they may prevent the establishment of the terrible habit for a few years; for after children enter their teens the susceptibility of an attack is almost nothing. The cause next in frequency to terror and sudden alarm is connected with the stomach, as eating some unaccustomed or indigestible article of food in large quantities.

We once knew a beautiful boy of promise, under ten, who having, with some companions got hold of some eggs, boiled them hard, and ate several, without anything else; he died in convulsions within a few hours. Often are our children on the verge of such results by the inattention of parents to their feeding; but they are relieved by spontaneous vomiting, bringing up a mass of sour, undigested food, perfectly nauseating, thus preventing fatal fever or the more terrible epilepsy. Bathing a child in cold water, after a hearty meal is quite sufficient to bring on an epileptic attack in a scrofulous constitution.

We were once called to an only child, about nine years old, in alarming convulsions, with incoherent utterances. He had eaten a hearty dinner, and from some childish freak had followed it up with an enormous amount of table salt. Nature would not vomit, but art gave instantaneous relief to an outraged stomach, and little Richard was himself again. Eating largely of soggy bread, or of the sodden undercrust of a pie, or of a pudding a little soured, may bring on an attack.

When an epileptic habit is once established, our main attention must be directed to avoiding the causes of attack and to the prevention of a threatened attack, waiting the meanwhile for one of those periods of life which are generally believed to make radical changes of constitution, either for better or worse; the most decided of which are the few years including fourteen and forty-two. One man represents that he prevents attacks in his own case by an iron wedge which he always carries about him; we should think a wooden one would answer the purpose, with greater convenience. As soon as he perceives a premonitory symptom, different in different persons, but present in all, and which a close observation will soon learn, he introduces it into his mouth so as to stretch it open to the utmost possible extent. The forcible distension or extension of any other muscle of the body would do the same thing, the pulling of a leg or arm, for example, but this requires the aid of another person; but, as everybody is often alone necessarily, it is important to have a remedy which the patient can apply himself promptly and at all times. Let any reader who is exempt from this infliction stop a moment in affectionate gratitude to Him who ruleth over all, that such a lot is not his own. It has been said that a black silk handkerchief thrown over the face while the fit is on, will bring the person "to" instantly.

No person subject to these attacks should ever be allowed to be alone or on horseback, or to walk along the banks of rivers, or in crowded streets, for obvious reasons. The attacks are sometimes indefinitely postponed by the most vigilant attention to diet.

While medicine has no power to cure epilepsy, it is very certain that grown persons can keep it in abeyance by the exercise of a close observation and a sound judgment, can, in other words, ward off an attack for a lifetime, by attention to two things: *First.* By avoiding, as to quantity and quality, the food which causes any kind of discomfort. *Second.* By regulating the system so as to have one full free action of the bowels every twenty-four hours. To look for restoration in any other direction is utterly hopeless. JOSEPH HEINRICH, M. D.

